# Structural Mechanisms of Forced Unfolding of Double-Stranded Fibrin Oligomers

**DOI:** 10.1021/acs.jpcb.5c00755

**Published:** 2025-04-14

**Authors:** Farkhad Maksudov, Anna D. Protopopova, Rustem I. Litvinov, Kenneth A. Marx, John W. Weisel, Valeri Barsegov

**Affiliations:** † Department of Chemistry, University of Massachusetts, Lowell, Massachusetts 01854, United States; ‡ Department of Cell and Developmental Biology, University of Pennsylvania School of Medicine, Philadelphia, Pennsylvania 19104, United States

## Abstract

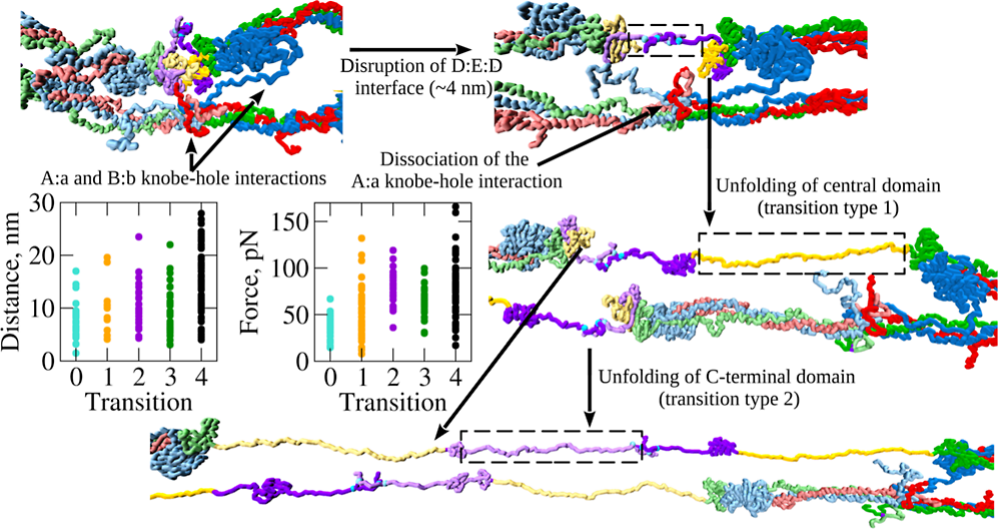

Fibrin forms a polymeric scaffold of blood clots, which are subjected to deformation in their dynamic environment. The extensible fibrin network allows fibers to stretch without breaking, but the mechanisms of their forced elongation are not understood. We combined atomic force microscopy, computer simulations, and Machine Learning to explore the nanomechanics of double-stranded cross-linked fibrin oligomers (FO). From the experimental force–extension profiles, the median 63 pN unfolding force and median 8.1 nm peak-to-peak distance with corresponding 56 pN and 11.4 nm interquartile ranges indicate substantial scatter due to ∼3–5 nm extension fluctuation of the triple α-helical coiled-coils. From simulations, unraveling of FO is determined by coupled dissociation of the D:D interface, γ-nodules unfolding, and reversible unfolding-refolding of the coiled-coils. These can occur as single structural transitions (60% of the time) or mixed transitions (40% of the time), with an alternating order of strands in which unfolding transitions occur, i.e., if the previous transition takes place in one strand, the next transition occurs in the other strand. The double-stranded FO are less extensible but stiffer and more stable compared with the single-stranded oligomers. These findings provide important insights into the biomechanics and dynamic structural properties of fibrin necessary to understand the (sub)molecular origin of fibrin extensibility.

## 1 Introduction

During blood clotting, fibrin forms a proteinaceous polymeric network that provides the mechanical scaffold of hemostatic clots that either beneficially prevent bleeding or can create pathological thrombi that obstruct blood vessels and impair blood flow. The fibrin network is a special type of extracellular matrix involved in various cellular reactions and biological processes, such as cell adhesion and mechano-transduction, wound healing and tissue regeneration, etc.^1,2^ In addition to its pathophysiological importance, over the past decades fibrin has been widely used as a versatile biomaterial in a variety of medical applications, e.g., as a hemostatic sealant, a substrate for tissue engineering and cell culturing, and a component of various targeted drug delivery systems.^3,4^

Insoluble fibrin is formed from a soluble plasma glycoprotein, fibrinogen, via limited proteolysis catalyzed by thrombin, a highly specific serine protease generated by the blood clotting enzymatic cascade. Fibrinogen is built of three pairs of polypeptide chains named Aα, Bβ, and γ that are linked together by disulfide bonds; the resulting polypeptide formula of fibrinogen is (Aα Bβ γ)_2_. Fibrin formation is initiated by the cleavage of four peptide bonds in fibrinogen followed by the release of two fibrinopeptides A (FpA) and then two fibrinopeptides B (FpB) from the N-termini of Aα and Bβ chains, respectively, to produce monomeric fibrin with polypeptide chain composition (α β γ)_2_. The cleavage of FpA leads to exposure of an N-terminal α chain motif Gly-Pro-Arg, called knob “A”, which binds with a high affinity to the constitutively exposed hole “a” in the globular γ-nodule of another fibrin molecule.^5,6^ The “A–a” knob-hole interactions are necessary and sufficient to initiate and drive polymerization of rod-like fibrin monomers, resulting in formation of double-stranded oligomers of varying length.^7,8^ Similarly, the cleavage of FpB exposes an N-terminal β chain motif GHRP, called knob “B”, which is complementary to hole “b” located in the β-nodule of another fibrin molecule. However, unlike the “A–a” knob-hole bonds, the weaker “B–b” knob-hole interactions are not necessary for fibrin polymerization, and they reinforce the lateral aggregation of protofibrils, making fibrin fibers thicker.^9^

Fibrin polymerization begins when two monomeric fibrin molecules bind through the “A–a” knob-hole interaction. Since a knob “A” is located in the central E-region and a complementary hole “a” is located on the lateral D-region, the rod-like molecules of fibrin bind to each other in a half-staggered fashion. The addition of a third fibrin molecule to the dimer occurs both through another pair of knob-hole bonds and linearly via an end-to-end association of the lateral globular D regions, such that two adjacent monomers form the D:D binding interface, i.e., an end-to-end junction between the monomers in one of the two strands in a fibrin trimer. New monomers add lengthwise and elongate each strand of the growing oligomer via formation of the transversal interstrand “A–a” knob-hole bonds and longitudinal intrastrand D–D interactions. This axial growth continues until the fibrin oligomers (FO) reach the length of two-stranded protofibrils ∼0.5 μm with 20–25 fibrin monomers, which undergo lateral aggregation to form fibrin fibers of varying width.^10^ The nascent fibers form branch points to create a 3D space-filling fibrous network comprising a macroscopic fibrin clot.^1^

The mechanical properties of the fibrin network determine the ability of hemostatic blood clots to stem bleeding at the site of injury and influence the course and outcomes of thromboses of various etiologies, including life-threatening diseases like heart attack and ischemic stroke.^11^ Due to the fundamental biological and medical importance of these pathologies, their underlying fibrin fiber and fibrin network biomechanics has become a rapidly developing field with a wealth of recent studies describing the structural basis of viscoelastic properties and rupture resistance of fibrin clots at various spatial scales spanning 7 orders of magnitude.^12−17^ One of the major aspects of fibrin mechanics is tensile deformation of the fibrin network, which implies compulsory elongation and molecular unfolding of fibrin molecules making up fibers as important mechanisms helping to accommodate mechanical stress. Possible molecular mechanisms underlying fibrins remarkable extensibility include forced unraveling of the triple-helical coiled-coils,^18,19^ unfolding of the globular γ-nodules,^20^ molecular straightening, especially in the unstructured and flexible αC regions,^21−24^ coiled-coils mechanical fluctuations, as well as more complex combinations of transitions.^14,17,25,26^ Despite a number of insightful studies, there is no agreement regarding the unfolding mechanism and (sub)molecular structures of fibrin fibers that undergo transitions in response to the stretching of a fibrin network.

**Figure 1:**
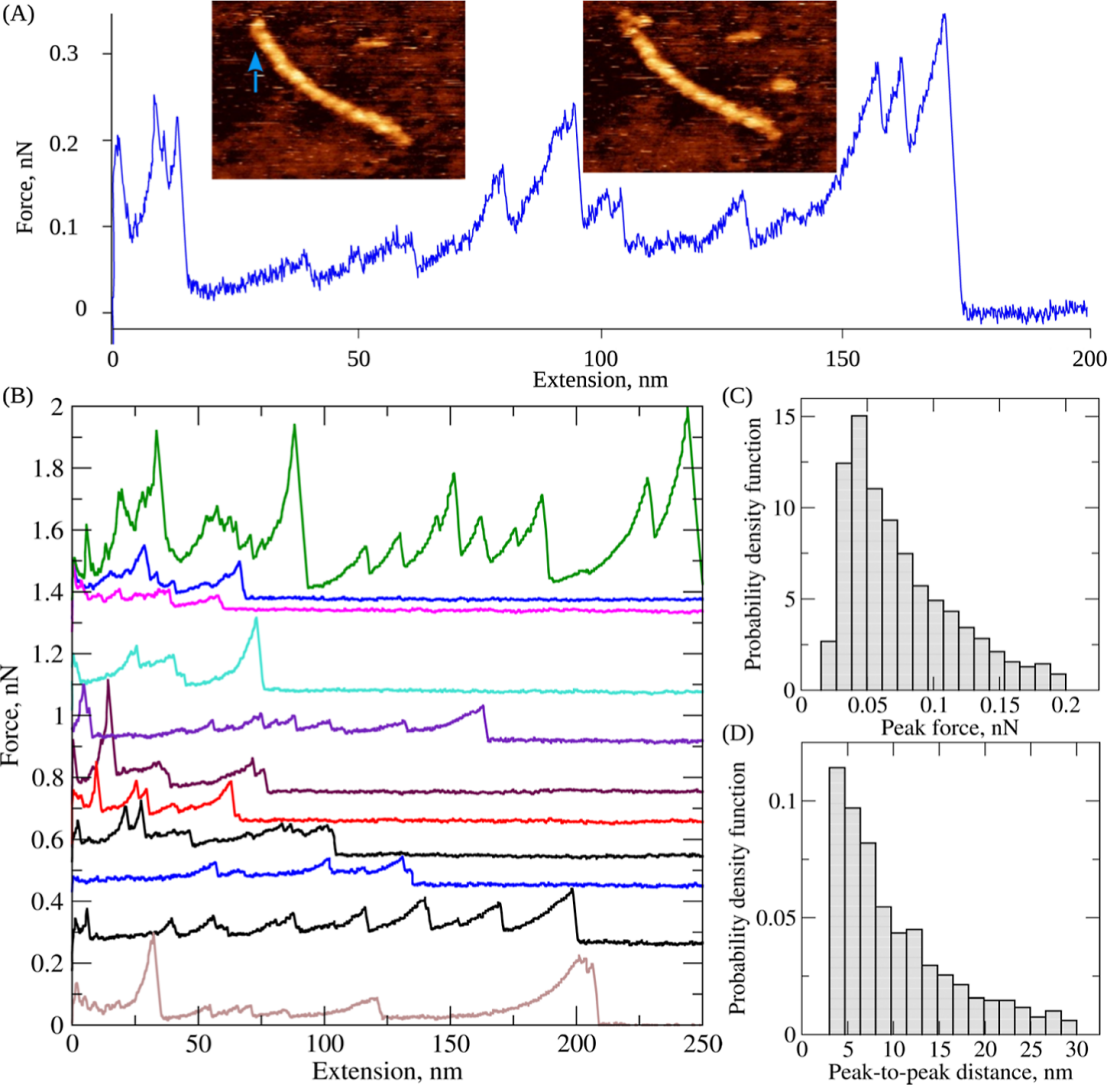
Experimental raw forced unfolding data for FO: Panel A: example of an experimental force–extension curve with large first desorption peaks, last tip detachment peak, and a sawtooth like pattern of unfolding force peaks in the middle. The insets are AFM images of a double-stranded fibrin oligomer FO4/3 taken before (left) and after (right) pulling force application, confirming the mechanical perturbation of the object. The arrow on the left image shows the point of force application. Panel B: Representative force–extension curves from AFM pulling experiments on forced unfolding of double-stranded FO of variable length, carried out using the pulling speed *v*_f_ = 0.2 μm/s. The differently colored force–extension (FX) curves are shifted along the *y*-axis for clarity. Panels C and D: The histogram-based estimates of the distributions of peak forces (unfolding forces) and peak-to-peak distances (chain extensions) were derived from experimental force–extension curves, selected as described in the Results section. In constructing the histograms, the bin size has been estimated using the Freedman-Diaconis rule for optimal bandwidth selection as described in ref (50).

Due to the large potential extensibility of a fibrin monomer^27^ (up to ∼450% of its 45 nm initial length with potential 250 nm total extension), the force-driven elongation of polymerized fibrin must involve unraveling of various compact molecular structures. Yet, the existing nanomechanical technologies [atomic force microscopy (AFM), optical trap, biomembrane force probe, etc.] do not allow investigators to resolve experimentally the structural transitions on the length scales of <1 nm.^28,29^ Furthermore, the complexity of fibrin(ogen) molecules (Figure 1) is overwhelming, and the variety of FO and polymers present at the same time makes it difficult (or even impossible) to uncover the unfolding mechanisms and resolve the unfolded structure using experiments alone. Computational biomolecular modeling—a powerful complementary approach to experiments, provides a detailed representation of submolecular structural rearrangements at the nanoscale. When coupled to protein forced unfolding experiments, this approach can be used to obtain structural fingerprints underlying the experimental force–extension profiles.^30^ The main challenge, however, is to follow computationally the protein unfolding dynamics on the experimental subsecond time scale using the physiologically relevant pico-to-nano-Newton force range. For these reasons, performing the full-atomic Molecular Dynamics simulations to study the mechanics of biopolymers of the size of fibrin having 3500 amino acid residues per fibrin monomer or ∼700,000 residues for an entire fibrin protofibril and stretched with the experimental 10^–1^–10^1^ μm/s pulling speeds is not computationally feasible. These limitations necessitate the development of simplified (or coarse-grained) models of proteins and the use of high-performance computing.

The Self Organized Polymer (SOP) model has been designed to describe the biomechanics of proteins at the C_α_-atom (amino acid) level of structural detail.^28^ We have implemented the SOP model on Graphics Processing Units (GPUs) to allow for the computational exploration of biological systems of large size (∼10^5^ amino acids) and to increase the timespan (centi-seconds) accessible in the simulations.^31−33^ These developments have been used to describe the biomechanics of large-size protein assemblies, including fibrin polymers, microtubules, cell organelles, and virus capsids.^34−38^ In our prior research on single-stranded FO,^29^ we combined AFM experiments and SOP-model based simulations to uncover the sequence of unfolding transitions in fibrin monomers forming single-stranded FO, which is a simpler structure to begin studies. The complex mechanism involves unraveling of the γ-nodules coupled to simultaneous unwinding and unfolding of the triple α-helical coiled-coils, resulting in formation of extended structures.^29^ Furthermore, the forced unfolding of the triple α-helical coiled-coils involves a transition in individual protein chains from the α-helical structure to the β-sheet structure,^39,40^ which was also verified experimentally.^41,42^ This remarkable α-to-β transition is an example of a phase transition in biological soft matter driven by the applied pulling force.^39,40^ Moreover, we demonstrated that the “A:a” knob-hole interactions, the strongest noncovalent bonds in fibrin polymerization,^43^ are characterized by the dynamic “catch-slip” transition.^44,45^ In a more recent study, we explored the mechanical properties and unfolding transitions in fibrin fibers formed by the un-cross-linked fibrin protofibrils (i.e., without covalent γ–γ cross-links).^46^

This paper advances the previously published work on the unfolding of synthesized or constructed computationally single-stranded fibrin(ogen) oligomers,^18,29^ with new AFM experiments, computer simulations, and statistical modeling^47^ now applied to the physiologically relevant cross-linked double-stranded FO (Figure 1). The experimental AFM force–extension profiles have been analyzed by comparing them with the protein unfolding measurements in silico performed under identical conditions of a mechanical force-ramp by using the structural models of FO and protofibrils constructed in our previous studies.^34,35^ The main findings reveal the interplay between the molecular elongation of FO, resulting in sequential unfolding transitions in the C-terminal γ chain nodules and reversible extension-contraction of the α-helical coiled-coil connectors in both strands. Interestingly, unlike in the single-stranded fibrin structures, the unfolding transitions in the double-stranded FO are characterized by an alternating order of strands, in which the unfolding transitions occur. These findings offer important insights into understanding the biomechanics and material properties of fibrin at the short oligomer and protofibril level of structure. The results obtained can be used more generally to describe the properties of other two- or multistranded polypeptide chain-based protein structures.

## 2 Materials and Methods

### 2.1 Formation of Fibrin Oligomers

To produce double-stranded FO, 0.15 mg/mL fibrinogen (Hyphen Biomed, France) in 20 mM Tris–HCl buffer (pH 7.4) containing 150 mM NaCl and 10 mM CaCl_2_ was mixed with 0.05 U/ml thrombin (final concentration), incubated for 10 min at room temperature, and immediately used in the AFM sample preparation as described below. To ensure covalent cross-linking of these fibrin structures, we added preactivated human factor XIIIa (Enzyme Research Laboratories, USA) at 6 μg/mL (final concentration) to the reaction mixture. The covalent cross-linking of FO was corroborated by the appearance of the γ-dimer band in SDS-PAGE of reduced samples (not shown).

### 2.2 Sample Preparation for Atomic Force Microscopy (AFM)

Microscope glass coverslips (Fisher Scientific, USA) were cleaned for 10 min by using PDC-32G-2 Plasma Cleaner (Harrick Plasma, USA). Initial protein solutions were diluted to 1–3 μg/mL concentration with 20 mM Tris–HCl buffer (pH 7.4) containing 150 mM NaCl and 10 mM CaCl_2_. Typically, 30 μL of a sample solution was applied on a glass surface, kept for 1–2 min, and then a 300–400 μL drop of the same buffer was placed above the sample. AFM imaging and pulling was performed in the drop of buffer.

### 2.3 AFM Experiments in the “Image-Pull-Image” Modes

AFM imaging and pulling of FO was performed in a physiological buffer using a MFP-3D microscope (Asylum Research, Oxford Instruments, USA) and short triangular cantilevers TR400PSA or TR400PB (Olympus, Japan) with a cantilever tip radius ≈20 nm. The cantilever spring constant (about 0.08 nN/nm) was calibrated prior to experiment by the thermal fluctuations method.^48,49^ We used an “image-pull-image” protocol consisting of the following three steps (Figure 1). First, we imaged the area of the sample in the tapping mode with a typical scan rate of 1 Hz to visualize and select an object (fibrin oligomer) for pulling. Next, we picked up a certain point on the object, attached a cantilever tip and performed several pulling-retraction cycles in the force mode to obtain sawtooth shaped force–distance curves, which reflect stretching and stepwise unfolding of the oligomer. The force–distance curves were acquired with the pulling speed of 0.2 μm/s and with a 5 s interval dwell in contact with the sample surface to enforce adhesion of the object to the cantilever tip. Peak forces exceeding 500 pN were considered too large and reflecting surface detachment events rather than representing protein unfolding; therefore, they were excluded from data analysis. After pulling, we reimaged the same area of the sample in order to see changes in the oligomer morphology and location ensuring its mechanical perturbation. Examples of AFM images of a fibrin oligomer before and after a pulling exercise, as well as the corresponding force–extension curve, are presented in Figure 1A. The bin size for the experimental histograms of the unfolding forces and peak-to-peak distances were calculated as described in ref (50). Meaningful force–extension curves (see Figure 1B for examples) have been selected based on the following selection criteria: (i) desirable last force peak (fibrin oligomer detachment from the cantilever tip) with at least two unfolding force peaks before the last force peak (the last force peak might be absent if the detachment force is comparable to the unfolding force); and (ii) repeated and reproducible pattern of the successive unfolding force peaks (force signals) that are well separated by the peak-to-peak distances resulting in sawtooth-like profiles of the unfolding forces versus fibrin oligomer extensions.

### 2.4 Self-Organized Polymer (SOP) Model of Covalently Cross-Linked Fibrin Oligomers

The mechanical properties of FO at the C_α_-atom-based amino acid level of detail were investigated using the native topology-based Self-Organized Polymer (SOP) model^28^ implemented in the SOP-GPU package.^31−33^ This model has been used to explore various large-size biomolecular assemblies, including: FO,^34,35,46^ microtubule filaments,^36,51^ virus shells, and cell organelles.^37,38,51,52^ In the SOP model, each amino acid is represented by its C_α_-atom, and the polypeptide chains of the double-stranded FO and protofibril are replaced by a collection of C_α_–C_α_ covalent bonds.^29,46^ The potential energy for a protein conformation *U_SOP_* is specified in terms of the coordinates of the C_α_-particles, {*r*_*i*_} = *r*_1_, *r*_2_,···,*r*_*p*_ (*p* is total number of amino acids) as *U_SOP_* = *U*_FENE_ + *U*_ANG_ + *U*_NB_^ATT^ + *U*_NB_^REP^. The finite extensible nonlinear elastic potential  describes the stretching deformations of the polypeptide chains. Here, *k* = 14 N/m is the spring constant for C_α–_C_α_ bonds, and *R*_0_ = 2 Å is the tolerance for the change in covalent bond distance. The summation in *U_FENE_* is performed over all covalently linked C_α_-particles and disulfide bonds, with *N*_bond_ representing the total number of covalent bonds in a polypeptide chain. The parameter *r*_b_ denotes the distance between the covalently linked particles, and *r*_b_^0^ is the equilibrium distance between these atoms in the reference structure (native folded state). In the bond-angle potential formed by two covalent bonds, , a constraint is imposed on the C_α_-particles separated by two covalent bonds (1–3 interactions) with the interaction energy range ε_ang_ = 4.2 kJ/mol and distance range σ_ang_ = 3.8 Å. The summation in *U*_ANG_ is performed over the N_ANG_ bond angles. To describe the noncovalent interactions that reinforce the native structure (i.e., native contacts), the Lennard-Jones potential  is used (ε_n_ is the interaction energy scale). The value of ε_n_ ranges from 2.5 to 5.4 kJ/mol, depending on the type of residue–residue contact (e.g., hydrophobic, electrostatic, etc.). A pair of residues forms a native contact if the distance between their C_α_-atoms is less than the cutoff distance *r*_c_ = 8 Å in the reference structure. The non-native contacts are described with the potential . Here, the summation is performed over all *N*_REP_ pairs of C_α_-particles that are neither covalently linked nor form a native contact; the values of strength ε_*l*_ and range σ_*l*_ of repulsion are set to 4.2 kJ/mol and 3.8 Å, respectively.

### 2.5 SOP Model-Based Pulling Simulations

**Figure 2:**
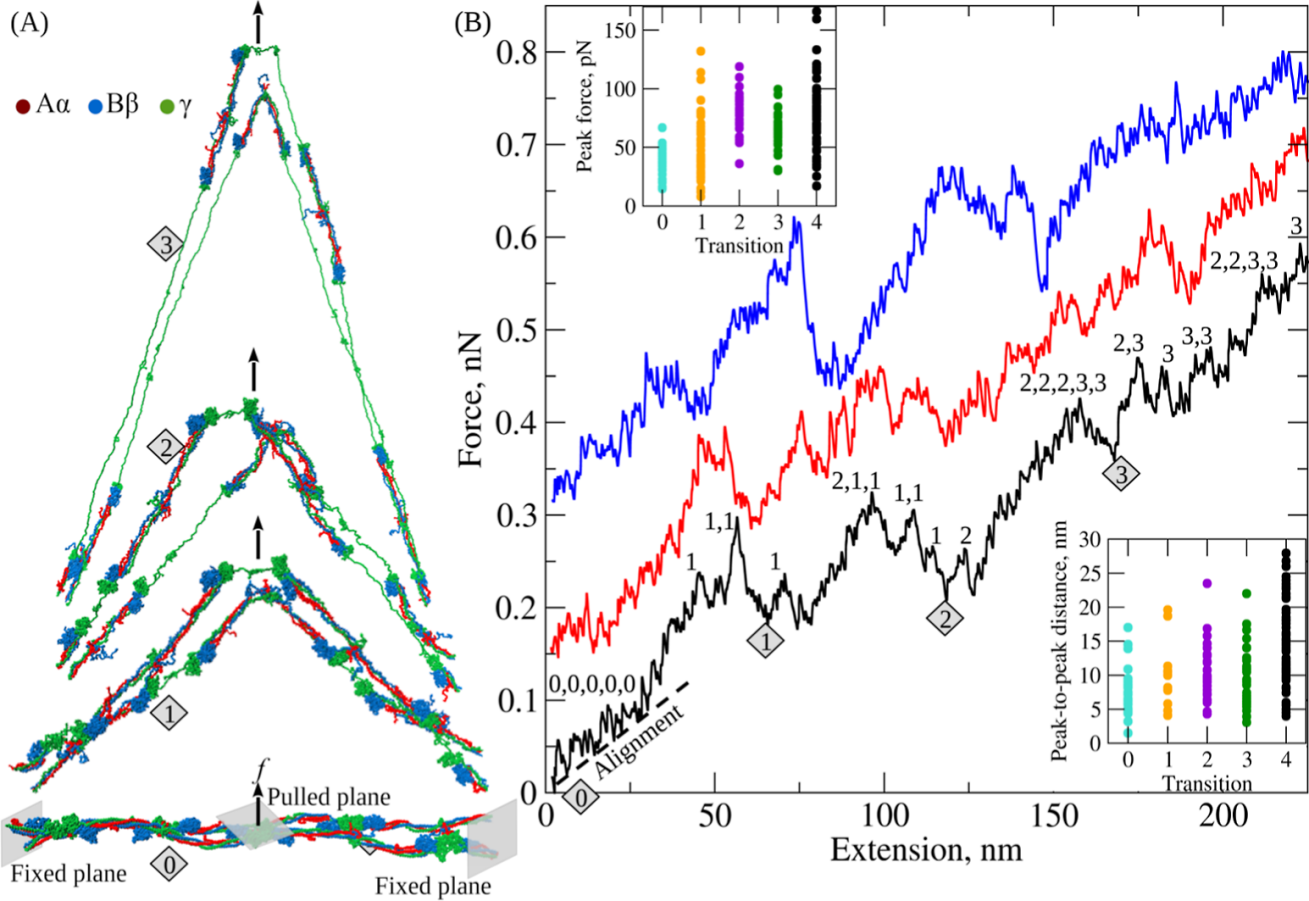
Unfolding of double-stranded fibrin oligomersin silico: Panel A: setup used in the pulling simulations (study 1) for fibrin oligomer FO3/2, in which the left and right ends of FO3/2 were constrained and the pulling force was applied to the central D–D interfaces (lower panel). Structure snapshots correspond to the folded state (snapshot 0), partially unfolded conformations (snapshots 1–2), and to the globally unfolded state (snapshot 3). These selected conformations (snapshots 0 to 3) correspond to the accordingly numbered regions in the black FX-curve in panel B. Panel B: representative force–extension curves for fibrin oligomer FO3/2 (shown in black, red, and blue color, and shifted along the *y*-axis for clarity) obtained from the simulations of forced unfolding performed with the 1.0 μm/s pulling speed. The force peaks corresponding to the structural transition types *j* = 0, 1, 2, 3, 4 are indicated above the force–extension curve shown in black color. The insets are scatterplots of the peak forces (top) and the peak-to-peak distances (bottom) corresponding to the transition types *j* = 0, 1, 2, 3, 4 for all FO combined. The dashed line at the beginning of the black FX-curve corresponds to the initial alignment and straightening (unbending) of the fibrin oligomer FO3/2 along the direction of pulling force.

To mimic the conditions of dynamic force ramp used in AFM experiments, in the simulations we constrain one part of the molecule (e.g., fibrin oligomer) and apply the pulling force to another part of the molecule. In the first set of simulations (study 1), both ends of the molecule are constrained, while the pulling force is applied to the central D:E:D interface (Figure 2A). Therefore, in study 1 residues αAsp114, βSer144, and γSer86 in the upper strand and residues αGln131, βAsn164, and γSer105 in the lower strand of the leftmost fibrin monomers and residues αArg118, βLys148, and γLeu90 in the upper strand and residues αIle127, βAsn160, and γLeu101 in the lower strand of the rightmost fibrin monomers are constrained. In the second set of simulations (study 2), the one (left) end of the fibrin oligomer is constrained, and the force is applied to the other (right) end (see Figure S2 in Supporting Information). In study 2, residues αAsp114, βSer144, and γSer86 in the upper strand and residues αGln131, βAsn164, and γSer105 in the lower strand of the leftmost fibrin monomers are constrained. The pulling force is exerted through virtual cantilever springs on residues αArg118, βLys148, and γLeu90 in the upper strand and residues αIle127, βAsn160, and residues γLeu101 in the lower strand of the rightmost fibrin monomers. We used the time-dependent force ramp *f*(*t*) = *r*_f_*t*, in which the force amplitude *f* increases over time *t* with a force-loading rate *r*_f_ = *k*_sp_*v*_f_ = 110 nN/s (*v*_f_ = 1 μm/s is the pulling speed and *k*_sp_ = 110 pN/nm is the virtual cantilever spring constant). The pulling simulations were performed, using the SOP-GPU package,^53^ on FO2/1, FO3/2, and FO4/4, formed by 2/1, 3/2, and 4/4 fibrin monomers in the first/second strand, respectively. The numerical output, i.e., structure of FO, coordinate, and energy files, were then analyzed. We have simulated 10 independent runs for fibrin oligomer FO2/1 (total time 0.8 s), 10 runs for FO3/2 (total time 2 s), and 5 runs for FO4/4 (total time 2 s). The pulling simulations were carried out using Brownian dynamics in the high-friction environment at room temperature (*T* = 298 K), , γ denotes the friction coefficient, and Γ_*i*_ represents the stochastic forces due to solvent corresponding to a water viscosity of 10^–3^ Pa × s.

### 2.6 Expectation-Maximization Method

In the Supporting Information, we outline in more detail the Expectation-Maximization (EM) algorithm for a pair of random variables sampled from the Gamma distributions, which are used to describe the skewed long-tailed probability distributions of the unfolding forces and peak-to-peak distances (see eqs S1–S27). Briefly, in the EM algorithm, we assume a bivariate Gamma distribution of the unfolding forces (*f*) and peak-to-peak distances (*x*), *p*(*f*, *x*; α, α′, β, β′, λ), and the prior probability **π** = {π_1_,···,π_*J*_} for each unfolding transition type *j* = 1,2,···,*J*.^47^ We define the mixture density *p* for the vector (*f*,*x*), which describes the unfolding force data *f* and the peak-to-peak distance data *x*. The application of the EM method relies on specifying initial values for the mean vectors of forces and distances, **μ** = {μ_1_,···,μ_*j*_} = {(μ_*f*1_,μ_*x*1_),···, (μ_*fJ*_,μ_*xJ*_)}, the standard deviations and covariances **σ** = {(σ_*f*1_, σ_*x*1_, σ_*fx*1_),···, (σ_*fJ*_, σ_*xJ*_, σ_*fxJ*_)}, and the prior probabilities **π**. These are converted to the shape and scale parameters for the Gamma distributions **α, α′, β, β′**, as well as parameter **λ** which controls the dependence between the forces and the distances (see eqs S12–S16 in the Supporting Information).

In the first expectation step, the logarithm of the likelihood (log-likelihood) of the expectation ln[*p*(***F***,***X***|**μ**,**σ**,**π**)] for test observations is calculated using the formula
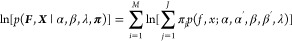
1In eq 1, ln[*p*(***F***,***X***|**μ**,**σ**,**π**)] is calculated using the estimates of the parameters describing the unfolding transition type *j* = 0, 1, 2, 3, and 4. Using the prior probabilities for each unfolding transition type *j*, π_1_,···,π_*J*_, the corresponding posterior probabilities γ_i_^j^ are calculated as
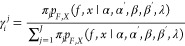
2In the second maximization step, new parameter values of the Gamma distribution μ_*fj*_, μ_*xj*_, σ_*fj*_, σ_*xj*_, σ_*fxj*_, π_*j*_, α_*j*_, α_j_′, β_*j*_, β_j_′, λ_*j*_ are calculated for each transition type *j* in order to maximize the log-likelihood ln[*p*(*F*,*X*|μ,σ,π)]. To calculate these quantities, we use the expressions for μ_*fj*_^new^, μ_*xj*_^new^, σ_*fj*_^new^, σ_*xj*_^new^, σ_*fxj*_^new^, π_j_^new^, λ_j_^new^, α_j_^new^, , β_j_^new^, and  accumulated in the Supporting Information (see eqs S17 and S18). The expectation and maximization steps are iterated until algorithm convergence is achieved. We defined the algorithm convergence as the point when the difference between the old and new values of the average unfolding forces is less than 0.1 pN, i.e., ∥μ_j_^new^–μ_j_^old^∥ < 0.1 pN, for all transition types *j* = 0, 1, 2, 3, and 4.

### 2.7 Analysis of Simulation Output

The histogram–based estimates of the probability density functions (distributions) of unfolding forces obtained from the dynamic force measurements *in vitro* and *in silico* were constructed using the Freedman–Diaconis rule for the bin size selection.^50^ To construct the model-free nonparametric estimates of the distributions of unfolding forces (Figures 1 and 4), we used the force (*f*) dependent kernel density , where *h* is the bandwidth and *f*_*i*_ is the *i*-th unfolding force observation, with the Gaussian kernel . We used Scotts rule^54^ to set the bandwidth *h* = *M*^–1/5^. To quantify the difference between the EM-based theoretical distributions of the unfolding forces corresponding to the experimental unfolding force data and the nonparametric densities of the unfolding forces obtained from the pulling simulations, we used the *L*^1^-norm
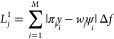
3In eq 3, *y*_*i*_ is the value of probability density function corresponding to the unfolding force *f*_*i*_, i.e., *y*_*i*_ = *p*(*f*) predicted by the EM algorithm, ψ_*i*_ is the value of the kernel density estimate corresponding to *f*_*i*_, i.e., ψ_*i*_ = φ_*K*_(*f*_*i*_), and Δ*f* is the force interval over which the density is constructed. In eq 3 above, π_*j*_ is the prior probability estimated theoretically and *w*_*j*_ is the weight of the *j-*th unfolding transition type estimated based on the results of pulling simulations.

## 3 Results

### 3.1 AFM Forced Unfolding Data

The fibrin structures of varying length cross-linked with factor XIIIa were allowed to adsorb on the atomically flat mica surface. The images of a short FO before and after pulling are presented in Figure 1A (insets), confirming the mechanical perturbation of the object. The initial imaging was followed by attaching a cantilever tip to a certain point on a FO and upward extension from the surface with subsequent registration of the resulting force–extension profile (Figure 1A). By repeating these measurements with many oligomeric fibrin structures, we were able to gather many hundreds of useful data traces of the force-induced extension and unfolding of FO. The force peaks reflect sharp increases in tension ramped up in the polypeptide chains forming the two-stranded FO due to pulling force application. These force peaks are followed by steep tension drops due to the unfolding transitions in FO, resulting in abrupt polypeptide chain slack.

To improve the quality of protein unfolding data collected in the AFM pulling measurements, in the subsequent data analysis, we only included the meaningful force spectra that pass the general selection criteria described in Materials and Methods, and that display the following characteristics: (i) minimal tip–surface interactions, providing a curve that has a typical sawtooth-like force–extension pattern with (ii) large initial and final force peaks resulting from fibrin oligomer detachment from the substrate surface or from the cantilever tip, respectively. Therefore, only the force–extension curves with a total extension exceeding 100 nm were selected to capture a comprehensive range of unfolding transitions in FO. The forced unfolding data obtained for short FO and longer oligomers (fibrin protofibrils) were combined together.

### 3.2 Selection of Peak Forces

Representative force–extension curves are displayed in Figure 1B, based on the selection criteria above. These force–extension curves were used to extract the unfolding force data obtained with the subtraction of baseline (i.e., force level to which the force drops following the force peak; see Figure S1 for illustration). The rationale for baseline subtraction is based on the following arguments: (i) elongation/unraveling of the coiled-coils in long rod-like fibrin monomers results in gradual tension increase in the double-stranded polypeptide chain; (ii) forced unfolding transitions that occur early on are characterized by lower (absolute) peak-force values compared to the unfolding transitions that occur later on (Figure S1); and (iii) the baseline in the simulated force–extension (FX) curves is higher than in the experimental curves, due to a higher pulling speed used in the simulations. Therefore, we subtracted from the peak forces the corresponding baseline reading. In addition, following our previous studies,^29^ we excluded forces >200 pN (over the baseline) from further analysis. The 200 pN threshold was chosen because it represents the maximum peak force observed earlier in AFM pulling experiments on fibrinogen monomers and single-stranded fibrin(ogen) oligomers,^29^ where coiled-coils contribute minimally to the unfolding force. Thus, the forces <200 pN after subtraction of the baseline values must reflect the realistic unfolding forces.

### 3.3 Selection of Peak-to-Peak Distances

Next, using our intuition described below, we excluded from subsequent analysis the unfolding data that displayed very long peak-to-peak distances (>30 nm). Indeed, an unfolding transition in one strand of a double-stranded FO leads to increased tension and an unfolding transition in the other strand, which starts unraveling before the unfolding transition in the first strand is complete, giving rise to a new peak at a distance shorter than a typical extension found in single-stranded oligomers.^29^ Differently stated, in a double-stranded oligomer, two unfolding events, one in each strand, are necessary to achieve the same extension as that found in a single-stranded oligomer. This results in shorter peak-to-peak distances observed in the double-stranded FO (see below), as compared to the single-stranded oligomers analyzed previously.^29^ Although simultaneous transitions, occurring in any combination and involving any number of transitions (see below), are expected to result in longer peak-to-peak distances, none exceeded 30 nm in our simulations. This value is lower than the maximum peak-to-peak distance observed in unfolding experiments on fibrin(ogen) monomers and single-stranded oligomers (explained below).^29^ Therefore, we applied a 30 nm cutoff to exclude longer peak-to-peak distances.

After carefully selecting the peak forces and peak-to-peak distances using the criteria justified above, we gathered the experimental unfolding data into the final data set, which we used for data analysis described below. The histogram-based estimates of the combined distributions of unfolding forces and peak-to-peak distances are displayed in Figure 1C,D, which show that these distributions are long-tailed. Analysis of the data revealed the median unfolding forces *f*_exp_ = 63 pN and the median peak-to-peak distances *x*_exp_ = 8.1 nm, with the interquartile ranges (IQRs) between the 25th and 75th percentiles of 56 pN and 11.4 nm, respectively. We also calculated the median value of the double-stranded chain stiffness *K* by evaluating the derivative (*K* = d*f*/d*x*) for the force–extension curves selected (i.e., average slope of the force peaks), and we found that *K*_exp_ = 13.5 pN/nm, with an IQR 11.8 pN/nm.

### 3.4 Forced Unfolding of Fibrin Oligomers In Silico

To provide a structural basis for the interpretation of the experimental forced unfolding data for FO, we turned to molecular modeling *in silico*. In the experiment, an AFM tip is lifting from the mica surface and stretching only a portion of the fibrin oligomer rather than the entire structure, so there is uncertainty about the size of the fibrin oligomer that has unfolded. For this reason, we carried out the forced unfolding experiments *in silico* on short two-stranded FO composed of 3 fibrin monomers (FO2/1), 5 monomers (FO3/2), and 8 monomers (FO4/4) in both strands (see Figures 2, S2, and S3). These structures were reconstructed in our previous studies.^34,35^ As was shown in a prior study,^55^ the αC regions do not interact with the fibrin protofibril or with each other within the protofibril. Furthermore, previous studies have shown that the absence of the αC regions does not qualitatively or quantitatively alter the characteristic sawtooth pattern of fibrin(ogen) unfolding.^29^ In pulling simulations on FO, the αC regions are not extended since tension does not propagate through these regions. Consequently, the αC regions were not included in the model.

In experiments, initially, the pulling force is applied in the direction perpendicular to the FO chains as the cantilever tip pulls and unfolds an FO from the substrate surface; but later, the direction of applied force becomes more and more parallel to the FO chain as the cantilever tip lifts and unravels the FO. To account for the changing geometry of force application realized in the AFM experiment, we used two different force protocols. In the first study, the pulling force is applied in the transverse direction perpendicular to the end-to-end vector of FO and it pulls at the middle portion of the FO (study 1; Figure 2); in the second study, the force is applied in the longitudinal direction coinciding with the end-to-end vector of FO and is pulled against the end of the constrained FO (study 2; Figure S2). These geometries of force application are shown in Figures 2A and S2A, respectively.

For study 1, we simulated a total of 25 independent trajectories of forced unfolding for FO2/1, FO3/2, and FO4/4 structures (4.8 s of total time; Figure 2A), and 25 more trajectories of forced unfolding for the same FOs for study 2 (11.8 s of total time; Figure S2A). For study 1, the representative simulated force–extension curves for FO3/2 are displayed in Figure 2B. The total extension, even for the smallest oligomer (FO2/1), is larger than 100–200 nm. This is because the FOs have bent initial conformations (snapshot 0 in Figure S2A), so in the simulations, the FOs first align along the direction of pulling force and straighten. As a result, the initial extension of FOs is due to their straightening, and only afterward do FOs begin to unfold.^46^ This explains why the initial 10–25 nm portions of the simulated force–extension curves for the FOs have a slope (Figures 2B and S2B), which also agrees with our published results.^46^ The simulated force–extension curves for the FOs of different lengths (FO2/1, FO3/2, and FO4/4) are similar. Using the simulated force–extension curves, we analyzed the force peaks corresponding to the total FO extension of *X* ≤ 200 nm, since this is the maximum extension observed in most of the experimental FX curves (Figure 1).

Analysis of the simulations from study 1 revealed the median unfolding force *f*_sim.1_ = 57 pN and the median peak-to-peak distance *x*_sim.1_ = 11.0 nm, with IQRs of 46 pN and 12.7 nm, respectively. For study 2, the median unfolding force is *f*_sim.2_ = 62 pN and the median peak-to-peak distance is *x*_sim.2_ = 17.0 nm, with IQRs of 43 pN and 13.3 nm, respectively. The median values of the FO chain stiffness are *K*_sim.1_ = 11.2 pN/nm and *K*_sim.2_ = 5.3 pN/nm with IQRs of 12.5 and 1.6 pN/nm, respectively. Interestingly, the median peak-to-peak distances from study 2 are more than twice as large than those observed in the experiments, which means that pulling the FO in the transverse direction (as in study 1), agrees better with the geometry of force application in the AFM experiment. Indeed, the statistics of unfolding forces and peak-to-peak distances from study 1 are similar to the statistics of experimental unfolding forces (*f*_exp_ = 63 pN) and unfolding distances (*x*_exp_ = 8.1 nm), and the difference is small, i.e., 6 pN and 2.9 nm. For study 1, the median FO chain stiffness is also close to the experimental value: *K*_sim.1_ = 11.2 pN/nm vs *K*_exp_ = 13.5 pN/nm vs *K*_sim.2_ = 5.3 pN/nm. These results indicate that the forced unfolding data (i.e., peak forces, peak-to-peak distances, and FO stiffness) generated in study 1 better correlate with the experimental results. Therefore, in the remaining part of this paper, we used the results from study 1 to interpret the experimental forced unfolding data.

### 3.5 Structural Outcomes of Forced Unfolding in Short Fibrin Oligomers and Protofibrils

Next, we performed extensive analysis and examination of the partially unfolded structures for FO2/1, FO3/2, and FO4/4 focusing mostly on short structures, such as pentameric FO3/2. We found that ∼60% of all of the unfolding transitions are single transitions that occur one after another in an alternating fashion in the two FO strands. That is, if the previous single transition has occurred in one strand, then the next single transition occurs in the other strand. The remaining 40% of unfolding transitions correspond to two (and occasionally three or more) transitions that always occur simultaneously, yet in different strands (mixed transitions). We found that the single transitions are characterized by a lower average unfolding force and shorter average peak-to-peak distance compared with the mixed transitions. For the mixed transitions, which always occur in two different strands, several distinct structural changes in different portions of the fibrin structure add up, resulting in one stronger force signal (rather than two separate weaker signals). Analysis of the results of simulations for FO2/1, FO3/2, and FO4/4 confirmed these findings about the existence of the single and mixed unfolding transitions. For FO2/1 and FO3/2, ∼80% of mixed unfolding transitions are combinations of only 2 transitions (i.e., one transition in each strand), whereas for FO4/4, ∼50% of mixed unfolding transitions are combinations of 4 transitions (i.e., two transitions in each strand).

**Figure 3:**
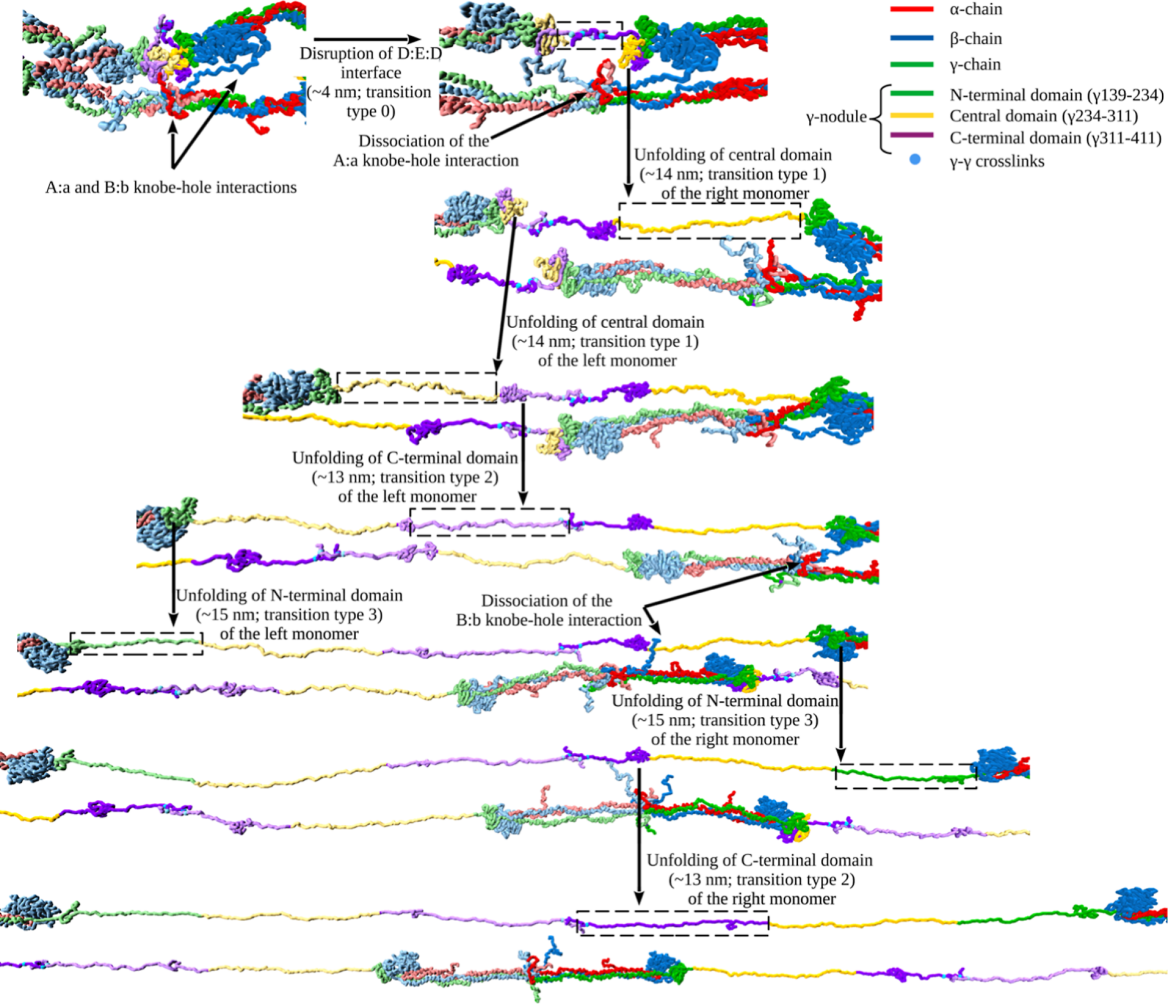
Forced unfolding of the γ-nodules forming the D:E:D interface in double-stranded fibrin oligomers in silico: Shown are the unfolding steps (transition types; Table 1) and intermediate structures observed for the γ-nodules. The dashed rectangles highlight parts of the protofibril where the unfolding transitions occur. Colors represent the α-chain (red) and β-chain (blue). The part of the γ-chain corresponding to the coiled-coil region (γ1–139), as well as the N-terminal domain of the γ-nodule (γ139–234), are shown in green. The residues corresponding to the central (γ234–311) and the C-terminal (γ311–411) domains of the γ-nodule are shown in yellow and purple, respectively. The γ-γ cross-linking sites (γ399 and γ406) are shown via cyan spheres. The adjacent monomers in the D:E:D interface are shown via different tones of the same colors. Also shown are the maximal extensions corresponding to the peak-to-peak distances observed in the force–extension curves.

Next, we characterized the structural transitions in FO using pentamer FO3/2 as an example. These transitions are depicted in Figure 2 and summarized in Table 1. Initially, the D–D interface undergoes a structural transformation from the “closed” conformation, in which the D–D junction is concealed, to the “open” conformation, in which the D–D interface is wide open (transition type 0; Figures 2 and 3; Table 1). This transition almost never gives rise to a detectable force peak, since it occurs at low <50 pN unfolding forces as a single transition (Figures 2B and 3) or at <65 pN unfolding force as a mixed transition in two different fibrin strands ({0,0}). Transition type 0 corresponds to a short 4.2 nm average extension (for single transition type 0), as compared to 8.4 nm average extension (for mixed transition {0,0}). This transition is almost always accompanied by the dissociation of both “A:a” knob-hole bonds and typically one of the two “B:b” knob-hole bonds (see Figure 3), while the other “B:b” knob-hole bond remains intact, because of the long 10.3 nm contour length of the N-terminal part of the β-chain. Next, the C-terminal β-strand (residues γ380–392) in the central domain of each γ nodule is pulled out,^56^ followed by the unraveling of residues γ234–311. This β-strand pullout triggers segregation of each γ nodule into two globular portions: one consisting of residues γ311–381, roughly corresponding to the C-terminal P-domain, and the other encompassing residues γ139–234, which includes the N-terminal domain. We collectively term these structural changes as transition type 1 (Figures 2B and 3; Table 1), which is characterized by a 50.9 pN average unfolding force and a 13.8 nm average chain elongation. This elongation stems from the unfolding of 89 residues in the γ chain and the partial unraveling of the coiled-coils. In transition type 2 (Figures 2B and 3; Table 1), with an 80.2 pN average unfolding force and a 13.1 nm average peak-to-peak distance, the C-terminal residues γ311–326 and γ339–380 in the γ nodule unravel. The chain elongation is a combination of the unfolding of 56 residues in the γ chain and partial extension of the coiled-coils. Finally, in the transition of type 3 (Figure 2B and; Table 1) with a 63.4 pN average unfolding force and a 15.2 nm average peak-to-peak distance, the N-terminal residues γ139–153 and γ182–234 of the γ-nodule unravel. The chain elongation arises from the unfolding of 66 residues and partial extension of the coiled-coils.

**Table 1:** Summary and Statistics of the Structural Transitions for Double-Stranded FO from Study 1: Summarized for Each Structural Transition of Type *j* = 0, 1, 2, 3, and 4 in Fibrin Monomers Forming the Oligomers are the Peak-to-Peak Distance and the Peak Forces (Relative to the Baseline) for the Single Transitions (Types 0, 1, 2, and 3) and for Simultaneous (Mixed) Transitions (Type 4)^a^

transition type	peak-to-peak distance, nm	peak force relative to the baseline, pN	structural changes
0	4.2 ± 0.1	36.3 ± 1.2	disruption of the D–D interfaces, dissociation of the “A:a” and “B:b” knob–hole bonds
1	13.8 ± 0.2	50.9 ± 0.7	Pull-out of the β-strand (γ380–392); unfolding of residues γ234–311; separation of the C-terminal part (residues γ311–380) and the N-terminal part (residues γ139–234); elongation of the coiled-coils
2	13.1 ± 0.5	80.2 ± 1.4	Unfolding of the C-terminal part of the γ-nodule (γ311–326 and γ339–380); elongation of the coiled-coils
3	15.2 ± 0.6	63.4 ± 1.6	Unfolding of the N-terminal part of the γ-nodule (γ139–153 and γ182–234); elongation of the coiled-coils
4	17.7 ± 0.1	80.1 ± 3.1	Any combination of transition of types 0–3, e.g.:{0,0},{1,1},{2,2,3,3},{1,2} etc.

a Shown are the average values and standard errors of the mean. Summarized are the main structural changes for each transition type.

Interestingly, each of the transitions of type 1–3 is followed by a ∼5–7 nm-long contraction (refolding) of the coiled-coil connectors. In the case of simultaneous (or mixed) transitions, which we refer to as transition type 4 (Table 1), any combination of two transitions of type 1–3 might occur at the same time. Examples are the mixed transitions {1,1}, {1,2}, {2,2}, {3,3}, {2,3}, etc. However, in our simulations, transition type 3 tends to occur at larger FO extensions after transition type 1 has occurred (see Figure 2B), which is why a combination {1,3} is rare. The average unfolding force for the transition type 4 is ∼80.1 pN and the average peak-to-peak distance is ∼17.7 nm. A summarized description of all of the structural changes and the statistics of the unfolding forces and the peak-to-peak distances corresponding to the transitions of type 0–4 for study 1 are presented in Table 1. A similar summary and statistical analysis of forced unfolding transitions for study 2 is provided in Table S1.

### 3.6 Machine Learning-Based Analysis of the Experimental Forced Unfolding Data

Although AFM experiments provide valuable information about the unfolding forces and elongations of the FOs, they cannot provide unfolding transition type-specific information. In addition, nonspecific system–substrate interactions, substrate desorption, cantilever tip detachment, uncertainty related to the length of FO portions that undergo unfolding, and the multitude of unfolding pathways, both single and mixed, necessitate using computational molecular modeling for the detailed interpretation of the experimental force–extension curves. Indeed, experimental histograms of the unfolding forces and peak-to-peak distances (Figure 1B) and the chain stiffness provide limited information about the unfolding transitions and unfolding pathways in FOs. On the other hand, computational models (such as the SOP model used here) might suffer from imperfections of the force field, faster pulling speeds (compared to those used in experiments), oversimplified force protocol, limited unfolding time span, etc. One can overcome these limitations by using a suitable statistical modeling technique, which is first trained by using the data from the simulations and is then applied to decipher the experimental distributions of forces and extensions.

The Expectation-Maximization (EM) algorithm (Materials and Methods) is founded on the idea that a large data set is composed of small subsets (for the transitions of type 0–4). The distribution of the combined unfolding data (i.e., combined distribution) is modeled as a weighted sum of the marginal distributions for these subsets.^47^ We applied the EM algorithm to resolve the marginal distributions of unfolding data for structural transition types 0–4 for FO using the combined distribution of the experimental unfolding data (Figure 1C). We set the initial values of the average quantities μ_***j***_ = {μ_*fj*_, μ_*xj*_}, standard deviations σ_***j***_ = {σ_*fj*_, σ_*xj*_}, and prior probabilities π_*j*_ (weights) for transitions of type *j* = 0, 1, 2, 3, 4 to be equal to the averages and standard deviation of unfolding forces (*f*) and peak-to-peak distances (*x*) estimated in study 1 (Table 1). First, we run the EM algorithm by repeating iteratively the expectation and maximization steps until algorithm convergence is achieved. The EM algorithm converged in less than 100 steps, which is a fast convergence. Next, we construct the marginal histograms and nonparametric densities of the unfolding forces ψ_0_(*f*), ψ_1_(*f*), ψ_2_(*f*), ψ_3_(*f*), and ψ_4_(*f*) for structural transitions of type *j* = 0, 1, 2, 3, and 4. Using eqs S12–S16 in Supporting Information, we convert the average forces μ_*fj*_ and the standard deviations σ_*fj*_ obtained with the EM algorithm to parameters of the Gamma distribution α_*fj*_ and β_*fj*_ and we substitute them into the marginal distributions *p*(*f*_*j*_|α_*j*_,β_*j*_) for the transitions of type *j* = 0, 1, 2, 3, 4. Finally, the combined theoretical distribution of the unfolding forces is obtained by calculating the weighted sum of the marginal distributions, i.e., *∑*π_*j*_*p*(*f_j_*|α_*j*_,β_*j*_), and is compared with the combined experimental distribution .

**Figure 4:**
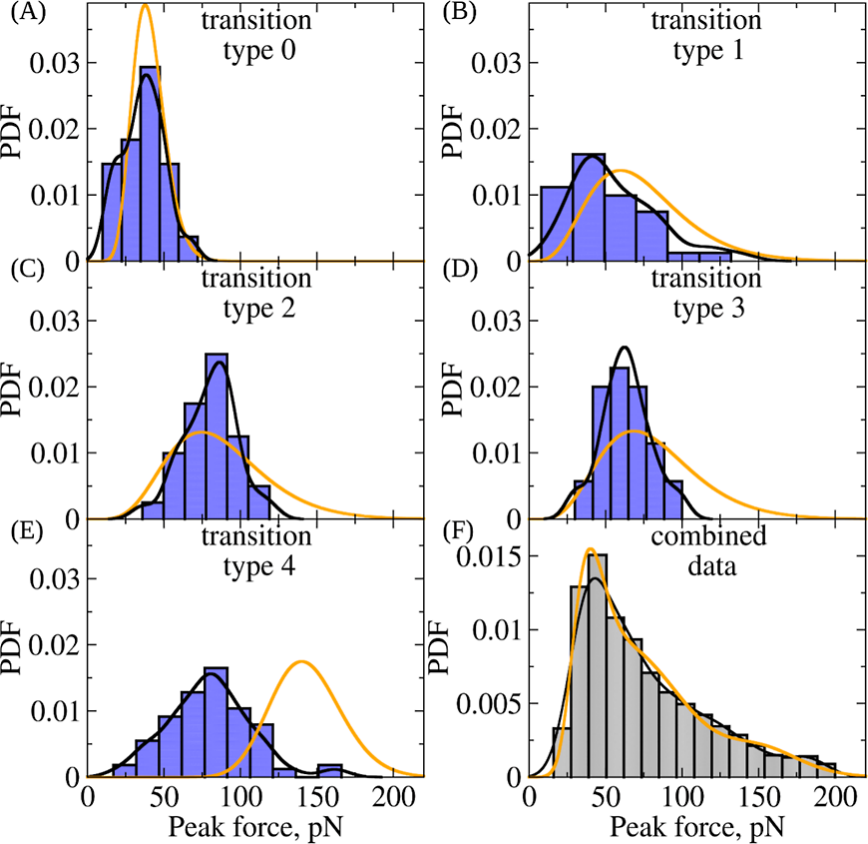
Performance of EM algorithm in modeling unfolding force data for double-stranded FO: Panels A-E show the histogram-based estimates (blue bars) and density estimates (continuous black curves) of the distributions of peak forces for different structural transition types 0–4 (Table 1) obtained from the simulations of forced unfolding (Figure 2). The EM-based theoretical density curves are shown as solid orange curves. Panel **F** shows the histogram-based estimate (gray bars; same as histogram in Figure 1C) and density estimate (black curve) of the combined distribution of unfolding forces (<200 pN) from single-molecule experiments on double-stranded FO for all the structural transitions combined. These are compared with the EM-based theoretical (solid orange) density curves. In constructing the histograms, the bin size estimation was carried out using the Freedman-Diaconis rule.^50^

We ran the EM algorithm to systematically vary π_*j*_, μ_*fj*_, μ_*xj*_, σ_*fj*_, σ_*xj*_, σ_*fxj*_, α_*j*_, α_*j*_′, β_*j*_, β_*j*_′,λ_*j*_. The final values of μ_*fj*_ and σ_*fj*_ are compared with the values of these quantities obtained from study 1 in Table 2. Because we used the results of simulations for μ_*fj*_ and σ_*fj*_, the EM-based predicted and simulated values can be directly compared. Although the algorithm utilizes bivariate Gamma distributions *p*(*f*, *x*), to jointly model the peak-to-peak distances *x* and the peak forces *f* (Figure 1C,D), we found that the average values of peak-to-peak distances are very similar across the transition types *j* = 0–4 (Table 1), resulting in considerable overlap among their respective peak-to-peak distance distributions. Consequently, we focused on the distributions of the peak forces (Figure 1C). In Figure 4A–E, we compare the marginal distributions of unfolding forces for transition types 0–4 from the simulations and from EM-based calculation. The theoretical combined distribution of the unfolding forces from the EM-based calculation is compared with the experimental combined histogram of unfolding forces in Figure 4F.

**Table 2:** Performance of EM Algorithm in Describing Unfolding Forces for Double-Stranded FO from Study 1: Compared for the Structural Transitions of Type 0, 1, 2, 3, and 4 are Theoretical Predictions for the Average Unfolding Forces μ_*fj*_, Standard Deviations σ_*fj*_, and Prior Probabilities π_*j*_ Obtained with the EM Algorithm^a^

methods/quantities	forced unfolding transition				
	type 0	type 1	type 2	type 3	type 4
μ_*fj*_, pN	42.3/36.3	68.4/50.9	91.4/80.2	96.4/63.4	141.0/80.1
σ_*fj*_, pN	11.9/13.6	28.2/28.5	30.9/17.8	30.7/16.2	21.4/27.9
π_*j*_	0.34/0.11	0.25/0.35	0.14/0.10	0.15/0.09	0.07/0.35
*L*_j_^1^	0.8	0.49	0.27	0.21	0.73

a The values of these same quantities obtained from the pulling simulations (study 1) are separated by a slash. Also shown are the corresponding values of *L*^1^–norm. The total error, *L*^1^–norm, for combined data is 0.08.

We found that for the structural transitions of type 0–4, the EM-based theoretical values of the average unfolding forces are 42.3 68.4, 91.4, 96.4, and 141 pN, respectively, while the average unfolding forces for the transitions of type 0–4 from the simulations are, respectively, 36.3 50.9, 80.2, 63.4, and 80.1 pN (Table 2). The standard deviations predicted by EM algorithm for distributions of transition types 0–4 are 11.9, 28.2, 30.9, 30.7, and 21.4 pN, respectively. From the simulations, the standard deviations of distributions of peak forces are 13.6, 28.5, 17.8, 16.2, and 27.9 pN, respectively. These numbers show that the EM model, first trained using the results of simulations and then applied to the experimental force unfolding data, helped to refine the unfolding force statistics for the structural transitions of type 0–4.

## 4 Discussion

Fibrin network is the main determinant of the mechanical properties of blood clots.^57−61^ While the mechanical and material properties of fibrin have been explored on multiple spatial scales from fibrin monomers to fibrin fibers, and to fibrin network using experiments and simulations,^14,29,46,59,60,62^ the molecular properties of two-stranded FO have eluded a detailed quantitative characterization because these reactive intermediate species rapidly form fibrin protofibrils, which in turn, aggregate laterally to form fibrin fibers. In our previous study, we found that the experimental sawtooth-like unfolding patterns in single-stranded FOs are due to reversible elongation-contraction of the fibrin coiled-coils and unfolding of the γ nodules in fibrin monomers.^29^ Therefore, it was important to compare the mechanisms of unfolding in the artificial single-stranded vs natural double-stranded FO, to identify similarities and to delineate any important differences.

In our previous studies, we developed multiscale modeling approaches that combine the atomic models and coarse-grained models of proteins to describe the mechanical properties of large-size protein assemblies.^31,32,39,40,43,46,51,53^ The computational acceleration on a GPU helps us to follow the dynamics of large protein structures (∼10^5–^10^6^ amino acids) at the C_α_-atom level of detail (<1 nm resolution) over a very long time scale (∼0.1 s) using the experimental force-loading conditions (10^–1^–10^1^ μm/s pulling speeds).^35,63^ The progress made has enabled us to explore the forced unfolding of fibrin monomers and single-stranded oligomers.^29^ In our prior study, we used the EM approach for classification of the protein unfolding data with a focus on single-stranded FO.^47^ In the current study, we extended the EM approach to characterize the unfolding transitions in two-stranded FO. While the molecular packing structure of fibrin fibers has become available from SAXS experiments,^64^ determination of the atomically detailed structures of FO cannot be accomplished by X-ray crystallography and/or electron microscopy. This is due to the thermodynamically unstable nature of these assemblies and their nonglobular elongated shape. In recent studies, we were able to construct the atomic structures of FO^34^ and fibrin protofibril^35^ using a clustering analysis of the crystal contacts displayed in resolved atomic structures of fibrin and its fragments. These oligomer structures were used in the present study.

We combined the AFM imaging and unfolding experiments with the C_α_-based coarse-grained molecular modeling and with Machine Learning, to explore the biomechanics and resolve dynamic structural changes in the two-stranded FO. We were able to image and manipulate these fibrin structures one at a time (Figure 1), and to probe their forced unfolding transitions using the AFM-based dynamic force-ramp technology. Using the experimental output (Figures 1 and 2), we selected meaningful force–extension curves with a characteristic pattern of repeated force peaks (Figure 1B), marking the unfolding transitions of compact domains in fibrin monomers, which were then statistically analyzed. The distributions of the unfolding forces and the peak-to-peak distances were found to be unimodal yet quite broad (Figure 1C,D), which indicates that in the FO a complex of unfolding reactions exists with many possible unfolding pathways, including single and multiple unfolding transitions. In this paper, which is a considerable advancement over our previous studies, we directly correlated the experimental and simulated forced unfolding data obtained for FO under the same force-loading conditions (rate and geometry of force application), to resolve the unfolded structures, and to uncover the unfolding mechanisms. Let us discuss the main findings in more detail.

### 4.1 Fibrin Oligomers Unfold through Structural Transitions in the γ Nodules and Dissociation of the D:D Interface

We observed the following five distinctly different single structural transitions, denoted as the transitions of types 0–4 (Table 1 and Figures 2 and 3). Transition of type 0 is the forced dissociation of the D–D interface, made up of the abutted ends of two fibrin monomers, which occurs under an unfolding force of ∼50 pN (Figure 2B), resulting in a short 4 nm extension. Transition of type 1 is the pullout of the C-terminal β strand in the central domain of the γ nodule (residues γ380 and 392), followed by the unraveling of residues γ234–311. In this transition, the γ nodule partially dissociates into two globular portions, the one consisting of residues γ311–381 (roughly corresponding to the C-terminal P-domain), and the other formed by residues γ139–234 (which include the N-terminal domain). This transition occurs under an average unfolding force of 50.9 pN and results in a 13.8 nm average extension. Transition of type 2 is the unraveling of C-terminal residues γ311–326 and γ339–380 in the γ nodule, which occurs with an average unfolding force of 80.2 pN and results in a 13.1 nm average extension. Transition of type 3 is the unraveling of the N-terminal residues γ139–153 and γ182–234 in the γ nodule at an average unfolding force of 63.4 pN and resulting in a 15.2 nm chain average extension.

All of these structural transitions 0–3 (Figure 3) make varying contributions to the overall elongation of the polypeptide chain (Table 1). Interestingly, these same transitions have been observed in our previous study, but for the single-stranded fibrin oligomer.^29^ However, the important difference between the results for the two-stranded vs single-stranded FO is that the peak-to-peak distances for the two-stranded FO are shorter due to geometric constraints on chain extensibility posed by the second strand. In the following discussion, we compare the results of this study with those obtained in our previous work.^29^ As the earlier study reported results using average values, we present the current results as averages as well rather than the medians reported in the Results section, which are different owing to the highly skewed distributions. A comparison of the experimental average values for the double-stranded FOs *x*_exp_ = 12.4 nm and single-stranded FOs (30.1 nm; ref (29)) demonstrates a 2.4-fold reduction (rather than the expected 2-fold). While the unfolding events in one strand trigger unfolding of the adjacent strand, a 2-fold decrease is not observed because in two-stranded FOs, we frequently observed simultaneous unfolding events that may occur as a few (typically two) events in each strand. In the current work, in the experimental pulling protocol, the pulling force was applied in the transverse direction (Figures 1A and 2A). Thus, effectively four strands of the double-stranded FO work together to sustain mechanical stress, since two shorter two-stranded portions of the same fibrin oligomer rather than one long single-stranded fibrin oligomer are stretched.^27^ Another important difference is that in the double-stranded FOs, mixed transitions might occur in four different strands simultaneously (transition of type 4), especially in longer fibrin fragments, thus decreasing the average peak-to-peak distance.

### 4.2 Role of Knob–Hole Bonds and Coiled-Coils in the Unfolding Mechanics of Fibrin Oligomers

The knobs “A” and “B” form noncovalent interactions between the two different strands in double-stranded FO. When mechanical tension develops in the two-stranded FO, short “A:a” knob-hole interactions dissociate because of the opening of the D:E:D interface (Figure 3), while longer “B:b” knob-hole interactions stay intact until the γ-nodule, where hole “b” is located, completely unfolds. To elucidate the role of “A:a” and “B:b” knob-hole interactions in forced unfolding of FOs, we created a virtual model of the FO2/1des-αβ fragment by removing the N termini of the α and β chains from fibrin oligomer FO2/1. We removed residues up to αAla27 in the α chain and residues βLys58 in the β chain, since these regions represent the most flexible segments of the N termini of α and β chains that are not resolved in the crystal structure of human Fg (PDB ID: 3GHG).^65^ We performed the pulling simulations on FO2/1des-αβ by applying the pulling force to one end of the fibrin fragment while constraining the other end (Figure S3). In this truncated fragment, the transitions of type 0 (disruption of the D:D interface) occur at a very short 3.2 nm extension vs 10.9 nm extension for FO2/1 (Figure S3). This means that in FO2/1des-αβ, the opening of the D–D interfaces occurs almost immediately. The average force for the transition type 0 is also lower: 33 pN for FO2/1des-αβ vs 43 pN for FO2/1. The other transitions (of types 1–3) are not affected by the absence of the “A:a” and “B:b” knob-hole in the FO2/1des-αβ variant (Figure S3). Therefore, the D:E:D interfaces are stronger by 10 pN in the presence of the stronger “A:a” knob-hole bonds as compared to the weaker “B:b” knob-hole bonds.^9^

Detailed structural analysis revealed an interesting role played by the coiled-coil connectors, namely, that reversible unfolding-refolding transitions help the coiled-coils to smooth out the effect of mechanical perturbation. When a pulling force is applied, the coiled-coil connectors gradually stretch and accumulate mechanical tension before and then release tension after the structural transitions have occurred. The coiled-coils are the most flexible portions of the fibrin monomer structure, which explains the quite large ∼4–5 nm coiled-coil length fluctuations. It can be concluded that the α-helical coiled-coils act as “molecular springs” in the deformation of fibrin. More generally, this mechanical action represents a new function of the α-helical coiled-coil structure of proteins. Another important point is that the β nodules in the fibrin monomers remain unaffected by mechanical tension and do not unfold. The reason being is that the mechanical tension does not propagate through the β nodules, since they are not engaged mechanically, and so they remain folded.

### 4.3 Double-Stranded Arrangement of Fibrin Monomers Increases the Stiffness of Fibrin Oligomers

By directly comparing the values of stretching stiffness for the double-stranded FO (analyzed in this work) with the values of the same quantity for the single-stranded FO (reported in ref (29)), we find that the inclusion of the second strand makes the two-stranded oligomers stiffer. Indeed, stiffness measured in the current experiment *K*_exp_ = 14.7 pN/nm is roughly 3-fold larger than for single-stranded FOs at a different geometry of pulling: K_exp_^ss^ = 4.9 pN/nm.^29^ We note that since in ref (29) we reported the average values, in this subsection we represent our findings in terms of the average values rather than using the medians as in the Results section. We used the medians in the Results section due to the highly skewed nature of the distributions of unfolding data (Figure 1C,D). However, this conclusion assumes that the experimental protocol in ref (29) involved a pulling geometry similar to that employed in the present study, specifically that the pulling force was applied in the transverse direction.

### 4.4 Double-Stranded Arrangement of Fibrin Oligomers Leads to Mixed Transitions and Alternating Order of Unfolding Events

Unlike the statistics of unfolding forces and peak-to-peak distances for the single-stranded FO,^29^ the distributions of these quantities for the double-stranded oligomers show long tails (Figure 1C,D). This is due to simultaneous transitions in the double-stranded oligomers, which result in longer peak-to-peak distances. In the single-stranded oligomers, the unfolding transitions occur one at a time, resulting in single force peaks. However, in the double-stranded oligomers, ∼40% of force peaks correspond to multiple or mixed (two, three, or more) simultaneous transitions. The longer the fibrin oligomer, the more frequently the transitions occur simultaneously, resulting in larger unfolding forces and longer peak-to-peak distances. We found that the early unfolding transitions are less likely to correspond to mixed transitions, while the later transitions are less likely to be single transitions. This is because the unfolding transitions that occur later in time (for total extensions >150 nm) are triggered by higher tension, and so there is a larger likelihood that several unfolding events occur simultaneously (Figures 2B and S2B), which also contributes to the larger peak-to-peak distances. In the experimental curves, however, the total extensions are shorter than 150 nm (Figure 1). This is because the total extensions >150 nm correspond to higher unfolding forces, at which FO dissociate from the substrate and/or the cantilever tip detachment occurs, which also explains why the experimental curves rarely exceed 200 nm total extension (Figure 1). These observations draw a molecular picture in which most of the fibrin oligomer structure remains attached to the substrate surface with only a small portion undergoing unfolding. This justifies our choice of using short FO3/2, rather than longer FOs in the simulations. Since there is no surface substrate in the simulations, the total extension even for the small oligomer FO3/2 (Figure 2B) is larger than the 200 nm extension observed in AFM experiment (Figure 1B).

The average peak force relative to the baseline for the single transitions is 55 pN vs 76 pN for the mixed transitions. This is because the average peak-to-peak distance for the single transitions (12.5 nm) is shorter than that for the mixed transitions (17.7 nm). According to Hooke’s law, force is proportional to extension (*f* = *kx*), so the larger peak-to-peak distance corresponds to a longer extension and results in the higher peak forces. During the single (mixed) transitions, one (several) domain(s) in one (both) strand(s) unravel, resulting in the shorter (longer) chain extension. Hence, the double-stranded arrangement of the polypeptide chains imposes geometric constraints, which also bring about the alternating ordering of the chains in which the unfolding transitions occur. If the previous transition had occurred in one strand, then the next transition would occur in the other strand. This is because the unfolding transition results in a sudden chain elongation and a corresponding tension drop in the strand where it has occurred; as a result, the next unfolding transition almost always occurs in the other strand, where tension is still high.

### 4.5 Geometry of Force Application Affects Unfolding Data Statistics but Not the Unfolding Mechanism

We compared the results observed for the same types of unfolding transitions in study 1 (Figure 2 and Table 1) and in study 2 (Figure S2 and Table S1). The average peak-to-peak distances for the single transitions of type 0–3 in study 2 are 5 nm for transition type 0, 19 nm for type 1, 16 nm for type 2, and 18 nm for type 3, respectively (Table S1). These numbers are consistently larger than the average values of 4.2, 13.8, 13.1, and 15.2 nm peak-to-peak distances for the transitions of type 0–3, respectively, observed in study 1 (Table 1). The average peak-to-peak distance for the mixed transitions (type 4) is also observed to be larger in study 2 (27.3 nm; Table S1) than in study 1 (17.7 nm; see Table 1). This is because in study 2 we apply pulling force to one end of a fibrin fragment, while constraining the other end (Figure S2). Hence, at the molecular level of detail, in study 2 we are pulling one longer two-stranded fibrin oligomer fragment (which contains two single fibrin strands; see Figure S2), whereas in study 1 we are pulling two shorter two-stranded FO (which contain four single fibrin strands; see Figure 2). Hence, in study 1, the unfolding transitions occur “more frequently”, because four shorter polypeptide chains are loaded mechanically, as compared to study 2, where the unfolding transitions occur “less frequently” with only two longer polypeptide chains loaded mechanically.

We compared the values of stretching stiffness from simulations for the double-stranded FO (studied in this work) with the values of the same quantity from simulations for the single-stranded FO (ref (29)). To facilitate direct comparison with the results of ref (29), here we present our results using the average values rather than the medians (as in the Results section). *K*_sim.2_ = 5.7 pN/nm (study 2), which is close to the stiffness for single-stranded oligomers (*K_sim_^ss^* = 6.0 pN/nm; ref (29) ). However, the changing geometry of force application results in a larger effect: *K*_sim.1_ = 15.9 pN/nm (study 1) is roughly 3-fold larger than stiffness for single-stranded FO at a different geometry of pulling *K_sim_^ss^*. Hence, changing the geometry of force application (effectively adding two more strands) makes FO significantly stiffer and more mechanically stable but less extensible. Furthermore, the unfolding data generated in study 1 correlate better with the experimental data because of the more similar geometry of force application realized in the simulations (study 1) and in the AFM pulling experiment. Hence, the geometry of the pulling force application is an important factor defining the mechanical properties of FO. Nevertheless, the same structural transitions of type 0–4 are observed both in study 1 and in study 2, implying that the geometry of pulling force application does not alter the mechanisms of unfolding of fibrin structures.

### 4.6 Differences between the Results of Experiment and Simulations

In AFM experiments, nonspecific interactions between the substrate surface and FO likely introduce noise into the experimental force spectra. This noise might come from the nonspecific binding/unbinding of the αC-regions or weakly adsorbed coiled-coil regions during pulling, which could generate the force signatures resembling partial unfolding events with the peak-to-peak distances below 5 nm and with the peak force below 50 pN. This results in a substantial number of low-force and low-peak-to-peak distance signals, which might resemble the peak forces and peak-to-peak distances associated with structural transitions of type 0 (disruption of the D:D interface). Consequently, the proportion (weight) of the unfolding transitions of type 0, π_0_, estimated using the EM algorithm based on the experimental data, is 0.34, compared to 0.11 found in the simulations. Also, the presence of peaks with low peak-to-peak distances contributes to minor discrepancies in the average peak-to-peak distance, with experimental data yielding an average value of 12.4 nm compared to the average value of 13.2 nm in the simulations. The poorest agreement between the experimental data (modeled with the EM algorithm) and the pulling simulations is observed for transitions of type 4. However, the agreement is better for transition types 1, 2, and 3 (Table 2), suggesting that these transitions are likely to occur with similar proportions (weights) π_*j*_, both in experiment and in simulations. The discrepancy for the unfolding transition type 4 may arise because in the simulations tension propagates through the coiled-coils to γ-nodules as the fibrin oligomers ends are fixed, whereas in experiments, the mica surface might overstabilize the D regions via adsorbed αC regions and other nonspecific interactions restricting conformational freedom and causing more unfolding transitions to occur simultaneously, thereby increasing the unfolding forces. In addition, interactions between mica and FO can transiently stabilize intermediate partially unfolded oligomer structures that are not observed in the simulations. These structures require additional force to disrupt the intraoligomer noncovalent interactions and adhesive nonspecific oligomer-mica contacts.

### 4.7 Significance of the Results of EM-Based Analysis

The results of the EM analysis quantitatively define the distinct unfolding transitions in FO. By utilizing marginal distributions of peak forces and related parameters, the EM algorithm effectively separates the complex experimental forced unfolding data into subsets, each representing specific unfolding pathways. The strong correlation between the EM-predicted and simulated average unfolding forces validates the algorithms accuracy in modeling the underlying physics of fibrin mechanical unfolding. Overlapping distributions of the peak-to-peak distances underscore the significance of peak forces as key descriptors of the structural transitions in FO. This methodology bridges experimental and computational findings, thus enabling detailed characterization of fibrin's mechanical properties and providing insights into the biophysical mechanisms governing its mechanical stability.

In conclusion, we have advanced the previously published work on the unfolding of single-stranded fibrin constructs,^18,29^ with new experiments, computer simulations, and statistical modeling,^47^ here applied to the natural double-stranded FO. These results are especially significant because these two-stranded oligomers are what are present in the fibers making up the network, providing the mechanical stability of blood clots and thrombi. We explored molecular unfolding via the low resolution experimental AFM force–extension profiles compared to the high-resolution protein unfolding simulations in silico, performed under identical conditions of the mechanical force-ramp by using the structural models of FO and protofibrils constructed in our previous studies.^34,35^ The simulations revealed the molecular structural identity of the transitions observed in the experimental force–extension profiles. These findings offer important mechanistic and structural insights above and beyond those provided by AFM measurements into understanding the biomechanics and material properties of fibrin at the fibrin oligomer level of detail. Since it has been demonstrated that forced unfolding occurs in clots after alignment of fibers along the direction of force,^24,25,40,41^ these insights are necessary to comprehend the effects of deformations of blood clots and thrombi from the forces present in the vasculature in vivo.

Going beyond the results obtained here, similar approaches can be used to describe the properties of other two- or multistranded polypeptide chain-based protein structures. Multistranded protein polymers, such as the double stranded actin filament,^66^ the meiotic synaptonemal complex SCP3 protein filaments,^67^ the collagen triple helix structure that forms the basis, via lateral aggregation, of structural collagen in the body,^68^ and the complex fibers formed by the long heterogeneously structured silk fibroin protein^69^ are all examples of complex structures that might benefit from application of the comparative AFM experiments and simulation of the forced unfolding we have described here. Going further afield, nonbiological multistranded polymeric structures might also be fruitfully studied using this approach.

## Supporting Information Available

A detailed description of the SOP model parametrization, formulation of the EM algorithm for the Gamma distribution, and force protocol used in the simulations in study 2; a summary and statistical analysis of the forced unfolding transitions for study 2; graphical representations of the baseline for unfolding force spectra; and the results obtained for double-stranded FO without “A:a” and “B:b” knob-hole bonds (PDF)
