# Supplementary material for: Structural Mechanisms of Forced Unfolding of Double-Stranded Fibrin Oligomers
Source: J Phys Chem B. 2025 Apr 14;129(16):3963–77. doi: 10.1021/acs.jpcb.5c00755 (PMC12035854; doi:10.1021/acs.jpcb.5c00755)
Supplement: Supplementary file 1 — jp5c00755_si_001.pdf [file jp5c00755_si_001.pdf]

Structural mechanisms of forced unfolding of double-stranded fibrin oligomers

Supplementary Information

Supplementary Methods

SOP Model parameterization: The SOP model parameterization for fibrin monomer is described in Ref. (28) of the main text. Briefly, we used the SOP models of fibrin oligomers composed of two, three, and four fibrin monomers in the upper strand and one, two, or four monomers in a lower strand, respectively, which form the fibrin oligomers FO2/1, FO3/2, and FO4/4. The native contacts were divided into the following six groups: 1) contacts in the central β–sheet of the γ–nodule (central B–domain), including residues γ189–197, γ243–284, and γ380–389 in the C–terminal β–strand (group 1); 2) contacts in the C–terminal part of the γ–nodule (γ284–380; group 2); 3) contacts in the N–terminal part of the γ–nodule (γ139–189 and γ197–243; group 3); 4) contacts in the α–helical regions in the coiled–coil connectors (α45–200, β76–197 and γ19–139; group 4); 5) contacts in the central nodule (α27–44, β58–75 and γ14–18; group 5) and 6) the rest of the native contacts, such as contacts between adjacent γ–nodules in D:E:D interfaces and inter-strand interactions (group 6). We set the values of ε_h to 2.9, 6.7, 5.0, and 5.4 kJ/mol for groups 1-3 and group 4-5, respectively. For group 6, ε_h was set to 4.2 kJ/mol (see Ref. (28) in the main text for more detail).

Formulation of EM algorithm for Gamma distribution: Let a random variable Y have a Gamma distribution with a shape parameter α and a rate parameter β. The probability density function for Y can be expressed as:

$$p_Y(\mathrm{y}) = \frac{y^{\alpha-1}e^{-\frac{y}{\beta}}}{\Gamma(\alpha)\beta^{\alpha}}; y > 0 \tag{S1}$$

Let another random variable Z have a Gamma distribution with a shape parameter α' and a rate parameter β'. The probability density function for Z can be expressed as:

$$p_Z(\mathrm{z}) = \frac{z^{\alpha'-1}e^{-\frac{z}{\beta'}}}{\Gamma(\alpha')\beta'^{\alpha'}}; z > 0 \tag{S2}$$

Assuming that variables Y and Z are independent, we can express forces (f) and peak-to-peak distances (x) as:

$$X = Y \text{ and } F = \lambda Y + Z \tag{S3}$$

where λ is some nonnegative constant parameter. Using Eqs. (S1) and (S2), the joint distribution of (Y, Z) can be expressed as:

$$p_{Y,Z}(y,z) = \frac{(y^{\alpha-1}e^{-\frac{y}{\beta}})(z^{\alpha'-1}e^{-\frac{z}{\beta'}})}{\Gamma(\alpha)\beta^{\alpha}\Gamma(\alpha')\beta'^{\alpha'}}; y > 0, z > 0 \tag{S4}$$

Assuming the inverse mapping $(y = x, z = f - \lambda x; x > 0, f > \lambda x)$ exists and using Eq. (S4), we can express the density of (F, X) as:

$$p(f, x; \alpha, \alpha', \beta, \beta', \lambda) = p_{Y,Z}\big(y(f,x), z(f,x)\big)|detA| \quad \text{(S5)}$$

where $A = \begin{pmatrix} \frac{\partial f}{\partial y} & \frac{\partial x}{\partial y} \\ \frac{\partial f}{\partial z} & \frac{\partial x}{\partial z} \end{pmatrix}$. The determinant of A is equal to 1, and so Eq. (S5) reduces to

$$p(f, x; \alpha, \alpha', \beta, \beta', \lambda) = x^{\alpha-1} e^{-\frac{x}{\beta}} \quad \text{(S6)}$$

The relationship between the parameters $\alpha, \beta, \alpha', \beta'$ and the moments μ_f, μ_x (means) and standard deviations and covariance $\sigma_f, \sigma_x, \sigma_{fx}$ can be expressed as:

$$\mu_x = E(\mathrm{Y}) = \alpha\beta \quad \text{(S7)}$$
$$\mu_f = E(\lambda\mathrm{Y} + \mathrm{Z}) = \lambda\alpha\beta + \alpha'\beta' \quad \text{(S8)}$$
$$\sigma_x^2 = \sigma_Y^2 = \alpha\beta^2 \quad \text{(S9)}$$
$$\sigma_f^2 = Var(\lambda\mathrm{Y} + \mathrm{Z}) = \lambda^2\alpha\beta^2 + \alpha'\beta'^2 \quad \text{(S10)}$$
$$\sigma_{fx}^2 = E(\mathrm{FX}) - \mu_f\mu_x = \lambda\alpha\beta^2 = \lambda\sigma_f^2 \quad \text{(S11)}$$

We can invert Eqs. (S7)-(S11) to obtain the following parameters of the joint Gamma distribution $p(f, x; \alpha, \alpha', \beta, \beta', \lambda)$:

$$\lambda = \frac{\sigma_{fx}^2}{\sigma_x^2} \quad \text{(S12)}$$
$$\beta = \frac{\sigma_x^2}{\mu_x} \quad \text{(S13)}$$
$$\alpha = \frac{\mu_x^2}{\sigma_x^2} \quad \text{(S14)}$$
$$\beta' = \frac{\sigma_f^2 - \lambda^2\sigma_f^2}{\mu_f - \lambda\mu_x} \quad \text{(S15)}$$
$$\alpha' = \frac{(\mu_f - \lambda\mu_x)^2}{\sigma_f^2 - \lambda^2\sigma_x^2} \quad \text{(S16)}$$

In the calculation of parameters μ_{fj}^{new}, μ_{xj}^{new}, σ_{fj}^{new}, σ_{xj}^{new}, σ_{fxj}^{new}, π_j^{new}, λ_j^{new}, α_j^{new}, $\alpha_j'^{new}$, β_j^{new}, and $\beta_j'^{new}$to maximize the log-likelihood $\ln[p(\boldsymbol{F}, \boldsymbol{X}|\boldsymbol{\mu}, \boldsymbol{\sigma}, \boldsymbol{\pi})]$, we use the following expressions:

$$\mu_{fj}^{new} = \frac{\sum_{i=1}^{M}\gamma_i^j f_i}{\sum_{i=1}^{M}\gamma_i^j} \quad \text{(S17)}$$
$$\mu_{xj}^{new} = \frac{\sum_{i=1}^{M}\gamma_i^j x_i}{\sum_{i=1}^{M}\gamma_i^j} \quad \text{(S18)}$$
$$\sigma_{fj}^{new} = \left[\frac{\sum_{i=1}^{M}\gamma_i^j (f_i - \mu_{fj}^{new})^2}{\sum_{i=1}^{M}\gamma_i^j}\right]^{1/2} \quad \text{(S19)}$$

$$\sigma_{xj}^{new} = \left[\frac{\sum_{i=1}^{M}\gamma_i^j(x_i-\mu_{xj}^{new})^2}{\sum_{i=1}^{M}\gamma_i^j}\right]^{1/2} \quad \text{(S20)}$$

$$\sigma_{fxj}^{new} = \left[\frac{\sum_{i=1}^{M}\gamma_i^j\left(f_i-\mu_{fj}^{new}\right)\left(x_i-\mu_{xj}^{new}\right)}{\sum_{i=1}^{M}\gamma_i^j}\right]^{1/2} \quad \text{(S21)}$$

$$\pi_j^{new} = \frac{\sum_{i=1}^{M}\gamma_i^j}{M} \quad \text{(S22)}$$

$$\lambda_j^{new} = \min[\frac{\sigma_{fxj}^{new^2}}{\sigma_{fj}^{new^2}}, \min_i(\frac{x_i}{f_i})] \quad \text{(S23)}$$

$$\alpha_j^{new} = \frac{\mu_{fj}^{new}}{\sigma_{fj}^{new^2}} \quad \text{(S24)}$$

$$\alpha_j'^{new} = \frac{(\mu_{xj}^{new}-\lambda_j^{new}\mu_{fj}^{new})^2}{\sigma_{xj}^{new^2}-\lambda_j^{new^2}\sigma_{fj}^{new^2}} \quad \text{(S25)}$$

$$\beta_j^{new} = \frac{\sigma_{fj}^{new^2}}{\mu_{fj}^{new}} \quad \text{(S26)}$$

$$\beta_j'^{new} = \frac{\sigma_{xj}^{new^2}-\lambda_j^{new^2}\sigma_{fj}^{new^2}}{\mu_{xj}^{new}-\lambda_j^{new}\mu_{fj}^{new}} \quad \text{(S27)}$$

Supplementary Table

Table SI. Summary and statistics of the forced unfolding transitions for double-stranded fibrin oligomers for Study 2: Summarized for each structural transition of type $j = 0, 1, 2, 3,$ and 4 in fibrin monomers forming the fibrin oligomers are the peak-to-peak distance and the peak forces (relative to the baseline) for the single transitions (types 0, 1, 2, and 3), and for the simultaneous (or mixed) transitions (type 4). Shown are the average values and standard error of the mean. Also, the main structural changes are summarized for each transition type.

Transition type	Peak-to-peak distance, nm	Peak force relative to the baseline, pN	Structural changes
0	5.0±0.1	55.0±0.1	Disruption of D-D interfaces, dissociation of the 'A:a' and 'B:b' knob-hole bonds
1	19±0.1	54.9±0.3	Pull-out of the β-strand (γ380-392); unfolding of residues γ234-311; separation of the C-terminal part (residues γ311-380) and the N-terminal part (residues γ139-234); elongation of the coiled-coils
2	16±0.2	49.1±0.6	Unfolding of the C-terminal part of the γ-nodule (γ311-326 and γ339-380); elongation of the coiled-coils
3	18±0.2	55.2±0.7	Unfolding of the N-terminal part of the γ-nodule (γ139-153 and γ182-234); elongation of the coiled-coils
4	27.3±0.2	70.4±0.3	It might be any combination of transition types, e.g.: {0,0}, {1,1}, {2,2,3,3}, {1,2} etc.

Supplementary Figures

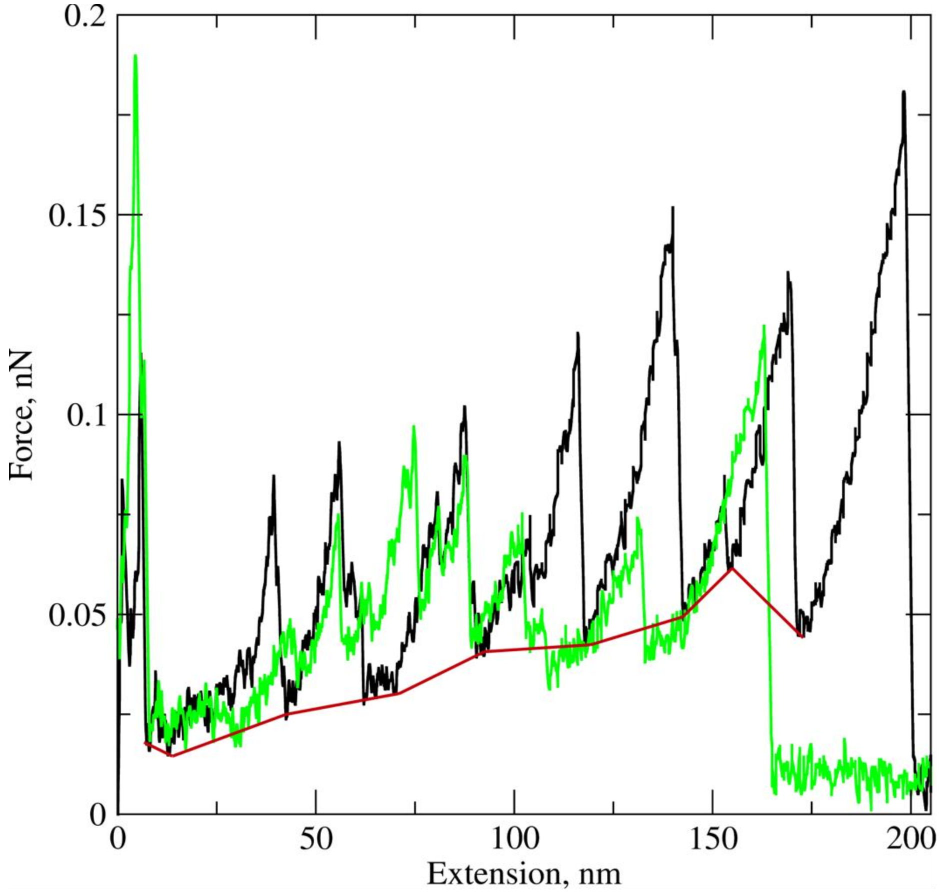

Figure S1. Graphical representation of the baseline for the unfolding force peaks: Shown in different black and green color are representative force-extension curves with the large first and last force peaks and with a typical saw-tooth like pattern of unfolding forces in the middle. The curves are characterized by the increasing magnitude of unfolding force minima and unfolding force maxima. The red line connecting all the force minima in the black force-extension curve exemplifies the baseline used in the estimation of the height of the force peaks corresponding to the real unfolding forces.

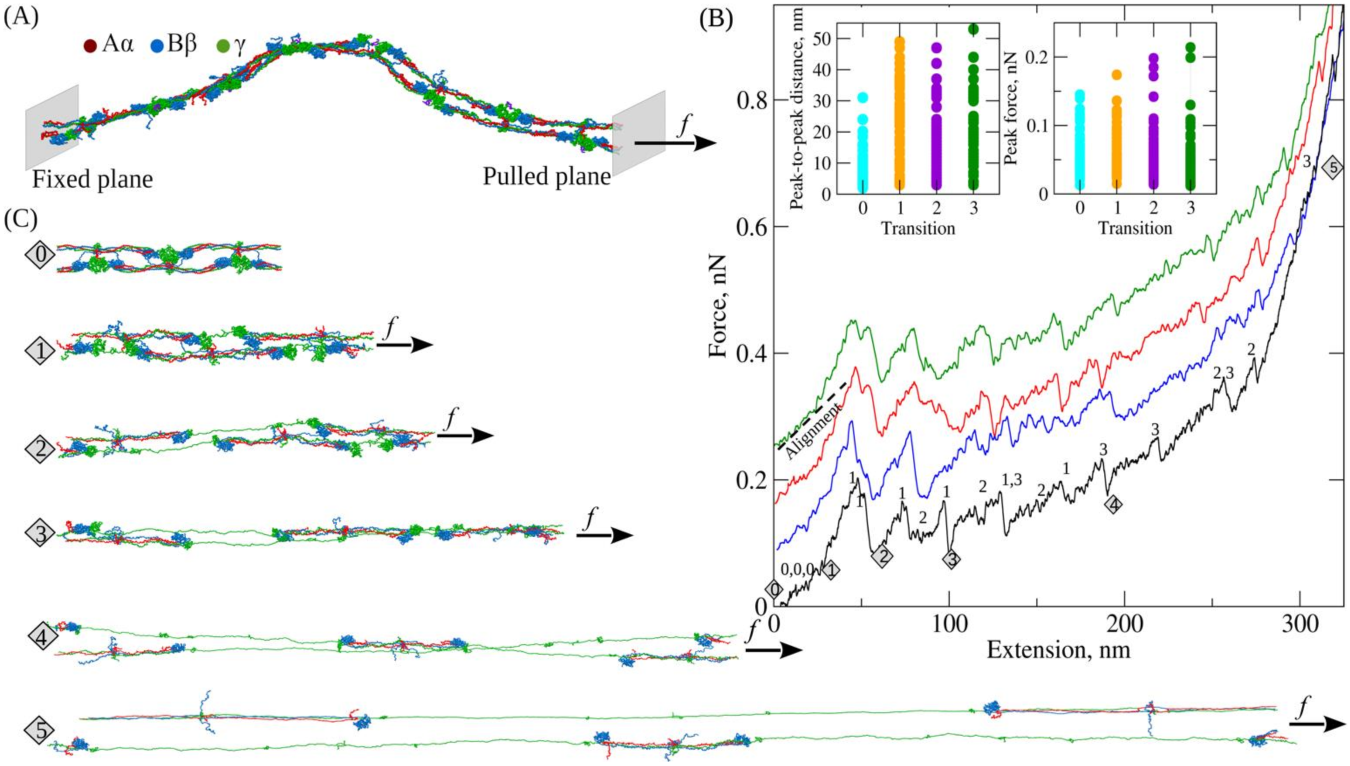

Figure S2. Unfolding of double-stranded fibrin oligomers *in silico*: Panel **A**: Force protocol used in the pulling simulations (Study 2) on fibrin oligomer fragment FO4/4. Here, the left end of the fibrin oligomer was constrained and the pulling force was applied to the right end of the fibrin oligomer (indicated by the black arrow). Panel **B**: Representative force-extension curves for fibrin oligomer FO2/1 (shown in black, blue, red and green color and shifted along the y-axis for clarity) obtained from the simulations of forced unfolding performed with a 1.0-μm/s pulling speed. The force peaks corresponding to the structural transitions of types $j = 0$, 1, 2, and 3 are indicated above the force-extension curve shown in black color. The dashed line in the beginning of the green FX-curve correspond to the initial alignment and unbending of the fibrin oligomer FO2/1. The *insets* demonstrate scatterplots of the peak-to-peak distances (left) and peak forces (right) corresponding to the transition types $j = 0$, 1, 2, 3 for all fibrin oligomers and protofibril combined. Panel **C**: Structure snapshots corresponding to the folded state (snapshot 0), partially unfolded conformations (snapshots 1-4), and the globally unfolded state (snapshot 5). These conformations (snapshots 0 to 5) correspond to the accordingly numbered regions in the black FX-curve in panel B.

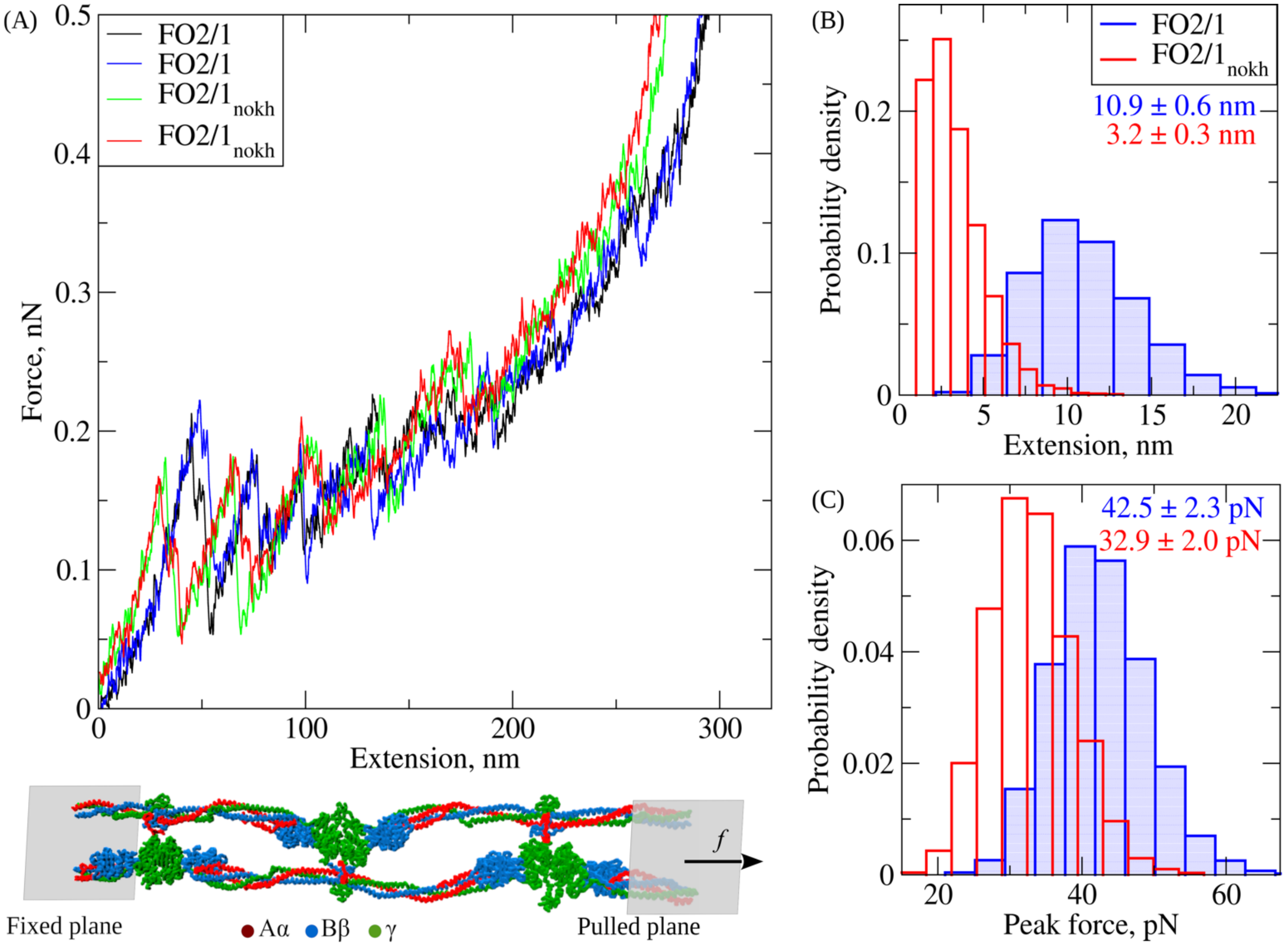

Figure S3. Unfolding of virtual constructs of double-stranded fibrin oligomers without 'A:a' and 'B:b' knob-hole interactions *in silico*: Panel **A**: Representative force-extension curves for fibrin oligomer FO2/1 with the 'A:a' and 'B:b' knob-hole interactions (black and blue curves) and for fibrin oligomer FO2/1_{nokh} without the 'A:a' and 'B:b' knob-hole bonds (red and green curves) obtained from the simulations of forced unfolding performed with the 1.0-µm/s pulling speed. The bottom panel depicts a force protocol used in the pulling simulations on fibrin oligomer FO2/1; here, the left end of FO2/1 is constrained, and the pulling force is applied to the right end of FO2/1. Panels **B** and **C**: The histogram-based estimates of the distributions of extensions and forces associated with the disruption of the D-D interface (transition type 0).
